# Contribution of rare and low-frequency whole-genome sequence variants to complex traits variation in dairy cattle

**DOI:** 10.1186/s12711-017-0336-z

**Published:** 2017-08-01

**Authors:** Qianqian Zhang, Mario P. L. Calus, Bernt Guldbrandtsen, Mogens Sandø Lund, Goutam Sahana

**Affiliations:** 1 Center for Quantitative Genetics and Genomics, Department of Molecular Biology and Genetics, Aarhus University, DK-8830 Tjele, Denmark; 2 Animal Breeding and Genomics, Wageningen University & Research, 6700AH Wageningen, The Netherlands

## Abstract

**Background:**

Whole-genome sequencing and imputation methodologies have enabled the study of the effects of genomic variants with low to very low minor allele frequency (MAF) on variation in complex traits. Our objective was to estimate the proportion of variance explained by imputed sequence variants classified according to their MAF compared with the variance explained by the pedigree-based additive genetic relationship matrix for 17 traits in Nordic Holstein dairy cattle.

**Results:**

Imputed sequence variants were grouped into seven classes according to their MAF (0.001–0.01, 0.01–0.05, 0.05–0.1, 0.1–0.2, 0.2–0.3, 0.3–0.4 and 0.4–0.5). The total contribution of all imputed sequence variants to variance in deregressed estimated breeding values or proofs (DRP) for different traits ranged from 0.41 [standard error (SE) = 0.026] for temperament to 0.87 (SE = 0.011) for milk yield. The contribution of rare variants (MAF < 0.01) to the total DRP variance explained by all imputed sequence variants was relatively small (a maximum of 12.5% for the health index). Rare and low-frequency variants (MAF < 0.05) contributed a larger proportion of the explained DRP variances (>13%) for health-related traits than for production traits (<11%). However, a substantial proportion of these variance estimates across different MAF classes had large SE, especially when the variance explained by a MAF class was small. The proportion of DRP variance that was explained by all imputed whole-genome sequence variants improved slightly compared with variance explained by the 50 k Illumina markers, which are routinely used in bovine genomic prediction. However, the proportion of DRP variance explained by imputed sequence variants was lower than that explained by pedigree relationships, ranging from 1.5% for milk yield to 37.9% for the health index.

**Conclusions:**

Imputed sequence variants explained more of the variance in DRP than the 50 k markers for most traits, but explained less variance than that captured by pedigree-based relationships. Although in humans partitioning variants into groups based on MAF and linkage disequilibrium was used to estimate heritability without bias, many of our bovine estimates had a high SE. For a reliable estimate of the explained DRP variance for different MAF classes, larger sample sizes are needed.

**Electronic supplementary material:**

The online version of this article (doi:10.1186/s12711-017-0336-z) contains supplementary material, which is available to authorized users.

## Background

Associations of common genetic variants with complex diseases and quantitative traits have been successfully identified in humans and livestock [1–3]. However, these loci explain only a small fraction of the total genetic variance of a trait. In human genetics, the portion of the additive genetic variance that remains unexplained by the associated genetic variants is known as the “missing heritability” [4–6]. One strategy to reduce the missing heritability is genomic prediction where all markers regardless of the magnitude and statistical significance of their effects are used to predict genetic values and estimate genetic variances [7–9]. Jensen et al. [8] reported that on average 77.2% of the genetic variance for six dairy cattle traits was attributed to genomic relationships constructed based on the Illumina BovineSNP50 BeadChip (50 k) single nucleotide polymorphisms (SNP)s. Román-Ponce et al. [7] reported that a genomic relationship matrix based on the 50 k SNP chip could explain between 51 and 94% of the genetic variance, depending on the reliabilities of the phenotypes used for milk yield, fat yield, protein yield and somatic cell count [1]. However, previous studies also showed that a wide gap remains between the proportion of variance explained using genomic relationships constructed from 50 k SNP chips and the genetic variance explained by pedigree-based relationships [7, 8, 10, 11]. This “missing” proportion of the genetic variance may affect the maximum accuracy that genomic prediction could achieve in livestock breeding [12].

Rare variants may play a significant role in quantitative trait variation [6, 13, 14] and contribute to the “missing heritability”. With the development of whole-genome sequencing technologies, next-generation sequence data have been generated for a large number of individuals in various cattle populations [15]. These sequence data have predominantly been used as a reference to impute SNP array genotypes to whole-genome sequences for individuals with phenotypes [16]. By using imputed sequence data, rare and low-frequency variants can be identified and studied for much larger numbers of individuals.

When whole-genome sequence data are available, linkage disequilibrium (LD) between SNPs and causal variants increases and a large fraction of the causal variants themselves will be available for analysis. Therefore, an increase in the proportion of the variance that can be explained for quantitative traits is expected when whole-genome sequence variants are used compared with the use of SNP array data [7, 8].

However, using whole-genome regressions which regress phenotypes on the whole-genome sequence variants using a linear model to infer the proportion of variance explained for a trait may result in biased estimates [17, 18]. First, if the causal variants are enriched in regions with higher or lower than average LD, heritability estimated based on genomic information is biased [1, 18]. Second, if causal variants have a different spectrum of minor allele frequencies (MAF) than the SNPs used, heritability estimated based on genomic information will also be biased [18]. Due to strong artificial selection, causal variants in dairy cattle are expected to often have extreme allele frequencies, whereas the content of DNA chips is biased by design towards highly polymorphic SNPs. Therefore, the spectrum of the allele frequencies of causal variants is expected to be quite different from that of SNPs on the commonly used 50 k chip. The effect of differences in the spectrum of allele frequencies and in LD heterogeneity on heritability estimates based on genomic information has not yet been studied in dairy cattle. However, several studies have shown that LD in bovine populations is relatively high, with long haplotype blocks, compared to that in human populations [19, 20]. Thus, we expect that the effect of heterogeneity in LD on heritability estimates is relatively small in bovine populations.

Recently, Yang et al. [18] proposed an LD- and MAF-stratified genomic-relatedness-based restricted maximum-likelihood (GREML-LDMS) method for human data that partitions the variance explained across classes of variants with different MAF. It also accounts for region-specific heterogeneity in LD [1]. They showed that heritability estimates obtained with the GREML-LDMS method were unbiased for human height and body mass index and found negligible missing heritability for both traits when using imputed variants [18]. Thus, we expect that, in cattle, the variance explained by imputed sequence data when estimated using the GREML-LDMS approach will capture larger proportions of the variance compared to estimates obtained from GREML using genomic relationships based on SNP chip genotypes [1, 21, 22].

The objectives of this study were to: (1) estimate the proportion of variance explained by whole-genome sequence variants for 17 traits in Nordic Holstein cattle; (2) estimate the proportion of variance explained by partitioning variants according to MAF, and with or without taking LD heterogeneity into consideration; and (3) compare estimates of the proportions of genetic variance explained by relationships based on pedigree, 50 k SNPs, and imputed whole-genome sequence variants.

## Methods

### Phenotypes and genotypes

In total, 5065 Holstein progeny-tested bulls with estimated breeding values were genotyped using the BovineSNP50 BeadChip (50 k) array version 1 or 2 (Illumina, San Diego, CA, USA). The phenotypes used in this study were deregressed estimated breeding values or proofs (DRP) with a minimum reliability of 0.2 for 17 traits (Table 1). For details regarding the 17 traits, recording procedures and models to estimate breeding values for these three indices, see http://www.nordicebv.info/ntm-and-breeding-values. The number of bulls with both genotype data and DRP for different traits ranged from 4485 to 4949 (Table 1).

**Table 1:** Description of the traits

Name of the trait	Abbreviations	Average DRP reliability	Standard deviation of DRP reliability	Range of DRP reliability	Number of bulls with DRP in the reference set
Yield index	YIELD	0.936	0.027	0.634–0.990	4649
Milk yield	MILK	0.934	0.031	0.634–0.990	4949
Protein yield	PROT	0.934	0.031	0.634–0.990	4876
Fat yield	FAT	0.933	0.031	0.634–0.990	4883
Udder index	MILKORG	0.773	0.080	0.444–0.990	4834
Milking speed	MILKSP	0.768	0.128	0.327–0.990	4753
Longevity	LONG	0.747	0.093	0.304–0.993	4551
Mastitis	MASTI	0.814	0.078	0.344–0.983	4858
Other-diseases (health)	HEALTH	0.577	0.132	0.207–0.990	4593
Feet and legs	LEG	0.570	0.121	0.204–0.990	4831
Daughter calving index (calving index)	CALV	0.670	0.090	0.204–0.990	4788
Service sire calving index (birth index)	BIRTH	0.738	0.083	0.442–0.990	4795
Fertility	FERT	0.671	0.112	0.214–0.990	4806
Body conformation index	BODY	0.805	0.071	0.513–0.990	4832
Growth	GROWTH	0.912	0.048	0.513–0.990	4397
Temperament	TEMP	0.603	0.135	0.212–0.990	4526
Nordic total merit index (NTM)	NTM	0.934	0.031	0.634–0.990	4834

DNA was extracted using standard procedures from either semen or blood samples. Genotyping was performed by GenoSkan A/S, Tjele, Denmark or the Department of Molecular Biology and Genetics in Aarhus University. The data editing steps were the same as in [23]. Quality parameters used to select SNPs were a minimum call rate of 85% for individuals and of 95% for loci. SNPs that were monomorphic or deviated from Hardy–Weinberg proportions (P < 0.00001) were excluded. The minimal acceptable GenCall score (GC) was 0.60 for SNPs and 0.65 for individuals. After quality control, 43,415 SNPs and 5065 individuals remained for analyses. The genomic positions of SNPs were taken from the UMD3.1 Bovine genome assembly [24].

In a previous study [23], the 50 k genotypes of 5065 animals were imputed to whole-genome sequence data using a two-step approach by first imputing 50 k genotypes to a high-density BovineHD BeadChip (HD, Illumina) using a multi-breed reference of 3383 animals, followed by imputing to the whole-genome sequence level using a multi-breed reference consisting of 1228 animals from run4 of the 1000 bull genomes project [15] and additional whole-genome sequences from Aarhus University [25]. The whole-genome sequence reference genotypes were pre-phased with BEAGLE4 r1274 [26]. Imputation to HD genotypes was done by using IMPUTE2 v2.3.1 [27] and imputation to the whole-genome level by using Minimac2 [28]. The imputed variants were filtered to remove those with a MAF lower than 0.001, which means that SNPs with less than ~10 copies of the minor allele in the data analysed were removed.

### Contribution of different classes of genetic variants based on MAF to DRP variance

**Fig. 1:**
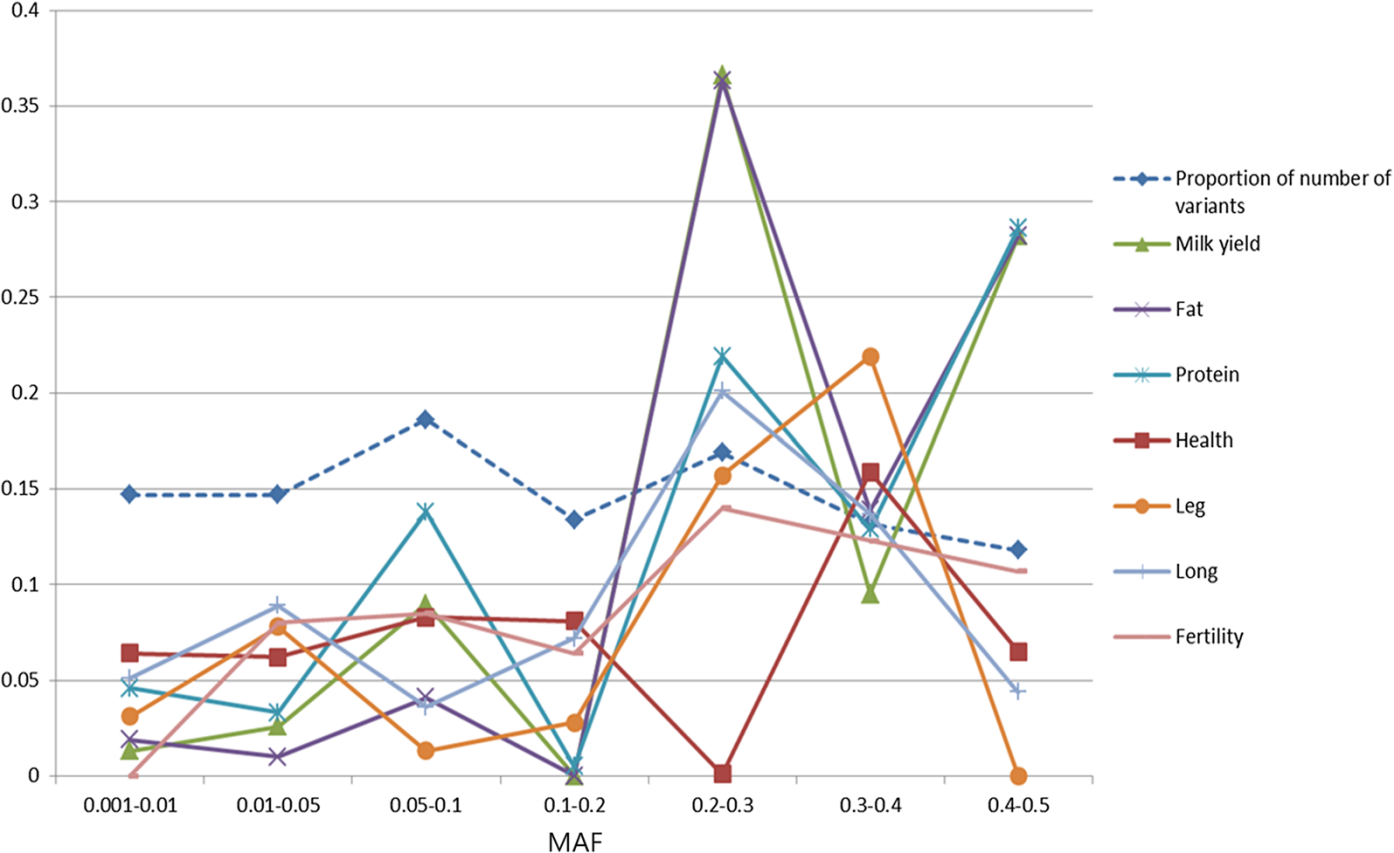
Proportion of variants in different MAF classes and their relative contribution to DRP variance for different traits. On the x axis are the variants with MAF classes: 0.001–0.01; 0.01–0.05; 0.05–0.1; 0.1–0.2; 0.2–0.3; 0.3–0.4 and 0.4–0.5 and on the y axis is the proportion of number of variants over the total number of imputed sequence variants (*dark blue*), relative contribution of explained DRP variance for different MAF classes variants for different traits based on “Trait abbreviation” in *different colors*

The GREML-MS and GREML-LDMS methods [18] were used to calculate the proportion of DRP variance explained by imputed sequence variants. For the GREML-MS method, the imputed sequence variants were grouped into seven classes based on their MAF (0.001–0.01, 0.01–0.05, 0.05–0.1, 0.1–0.2, 0.2–0.3, 0.3–0.4 and 0.4–0.5). The number of variants was very similar across MAF groups (Fig. 1). Rare variants were defined as those with a MAF ranging from 0.001 to 0.01; low-frequency variants as those with a MAF ranging from 0.01 to 0.05; and common variants had a MAF higher 0.05. Average imputation accuracies (IMPUTE-INFO score defined by [29]) for rare and low-frequency variants were 0.850 and 0.873, respectively [see Additional file 1: Table S1]. We did not filter variants strictly based on imputation accuracy, i.e. all variants with IMPUTE-INFO score were included in the analyses, because a study using human data suggested that removing variants based on a more restrictive IMPUTE-INFO threshold leads to a loss of variance explained [18].

Genomic relationship matrices (GRM) for each of the seven classes of variants were calculated following [1] and fitted jointly in a multicomponent REML analysis:

1 $${\mathbf{y}} = {\mathbf{1}}\mu + \mathop \sum \limits_{i = 1}^{7} {\mathbf{g}}_{i} + {\mathbf{e}}\text{,}$$

where ${\mathbf{y}}$ is the vector of phenotypes (DRP), **1** is a vector of 1s, $\mu$ is the general mean, ${\mathbf{g}}_{i}$ is a vector of the genetic values for the $i$th variant class ($i$ = 1,2,…,7), ${\mathbf{g}}_{i} \sim{\text{N}}\left( {{\mathbf{0}},{\mathbf{G}}_{i}\upsigma_{i}^{2} } \right)$, where ${\mathbf{G}}_{\text{i}}$ is the GRM of the $i$th class, and ${\mathbf{e}}$ is a vector of residuals with ${\mathbf{e}}\sim{\text{N}}\left( {{\mathbf{0}},{\mathbf{I}}\upsigma_{e}^{2} } \right)$. The variance components were estimated by using the REML approach implemented in the genome-wide complex trait analysis (GCTA) software [30, 31]. The proportion of variance in DRP explained by class $i$ of variants was calculated as:

$${\raise0.7ex\hbox{${{\hat{\sigma }}_{i}^{2} }$} \!\mathord{\left/ {\vphantom {{{\hat{\sigma }}_{i}^{2} } {\mathop \sum \nolimits_{i = 1}^{7} {\hat{\sigma }}_{i}^{2} + {\hat{\sigma }}_{e}^{2} }}}\right.\kern-0pt} \!\lower0.7ex\hbox{${\mathop \sum \nolimits_{i = 1}^{7} {\hat{\sigma }}_{i}^{2} + {\hat{\sigma }}_{e}^{2} }$}}.$$

To account for the region-specific heterogeneity in LD, we used the GREML-LDMS approach proposed by Yang et al. [18]. First, for each SNP, an LD score was computed as the sum of the LD measure r^2^ between this SNP and other SNPs in a 20-Mb region centered on this SNP. Then, the mean LD score of the variants in each segment which contained twice the average number of variants per 100-kb window of a chromosome was calculated and these were used to partition the variants within each of the seven MAF classes into four equally-sized LD groups based on increasing mean LD scores, following Yang et al. [18], resulting in 28 groups. Then, Model (1) was fitted using these 28 genetic components. In addition, to compare the estimates of variance components based on the GREML-MS and GREML-LDMS methods, the variants were also stratified into three different LD groups within each of the seven MAF classes, resulting in 21 genetic components. The proportion of DRP variance explained by rare, low-frequency and common variants, as defined previously, was divided by the sum of the DRP variances to compare their relative contribution to the total DRP variance explained.

The GRM used in GCTA, assumes that allelic effects of both common and rare variants follow the same distribution, similar to VanRaden’s method 2 [21, 32]. This means that a common variant explains more variance than a rare variant. To verify whether this assumption is reasonable, expected contributions of different classes of MAF variants to the variance were compared to our empirical results. The expected variance explained by the variants from different MAF classes were computed under the assumptions of VanRaden’s methods 1 and 2 [21]. For VanRaden’s method 1, the expected variance explained by a class of variants is:

$$\mathop \sum \limits_{i = 1}^{{j_{1} }} 2p_{i} \left( {1 - p_{i} } \right)/\mathop \sum \limits_{i = 1}^{{j_{2} }} 2p_{i} (1 - p_{i} ),$$

where $p_{i}$ is the MAF of the $i$th locus and the numerator is the sum for the variants in each class until the $j_{1}$th locus and the denominator is the sum for all the variants until the $j_{2}$th locus. For VanRaden’s method 2, the expected proportion of genetic variance explained by a class of variants is $N_{class} /N$, where $N_{class}$ is the number of variants per class, and $N$ is the total number of loci used in the calculation. Correspondingly, VanRaden’s method 1 assigns a large amount of variance to common variants, while VanRaden’s method 2 puts more emphasis on rare variants.

The phenotypes used in our analysis, as is often the case in animal breeding, were DRP derived from estimated breeding values with varying reliabilities. Weights derived from those reliabilities are commonly used in analyses that use DRP. However, the GCTA software does not support the use of weights, because it was developed in the context of human data analysis where the phenotypes used are typically directly measured on the genotyped individuals. However, the average reliability of the DRP used here were quite high (Table 1). For example, the average reliability of milk yield was 93.4%. Therefore, ignoring DRP reliabilities in our analyses is not expected to affect the results.

### Proportion of DRP variance captured by pedigree and 50 k SNPs

The genetic variance estimated by using the pedigree relationship matrix was compared to the variance explained by the imputed sequence variants and the 50 k SNPs. The proportions of DRP variance explained by pedigree and genomic relationships were estimated by fitting pedigree and 50 k data separately or jointly in the model as described below:

2 $${\mathbf{y}} = {\mathbf{1}}'\mu + {\mathbf{Z}}_{a} {\mathbf{a}}_{1} + {\mathbf{e}},$$

3 $${\mathbf{y}} = {\mathbf{1}}'\mu + {\mathbf{Z}}_{g} {\mathbf{g}}_{1} + {\mathbf{e}},$$

4 $${\mathbf{y}} = 1'\mu + {\mathbf{Z}}_{g} {\mathbf{g}}_{2} + {\mathbf{Z}}_{a} {\mathbf{a}}_{2} + {\mathbf{e}},$$

where ${\mathbf{y}}$ is the vector of phenotypes, **1** is a vector of 1s, $\mu$ is the general mean. ${\mathbf{Z}}_{g}$ and ${\mathbf{Z}}_{a}$ are incidence matrices that relate DRP to breeding values in ${\mathbf{g}}_{1}$, ${\mathbf{g}}_{2}$, ${\mathbf{a}}_{1}$ and ${\mathbf{a}}_{2}$, respectively. Vectors ${\mathbf{a}}_{1}$ and ${\mathbf{a}}_{2}$ contain random effects with variance ${\text{var}}\left( {\mathbf{a}} \right) = {\mathbf{A}}\sigma_{a}^{2}$, where ${\mathbf{A}}$ is the additive genetic relationship matrix computed from pedigree records. Finally, ${\mathbf{e}}$ is a vector of residuals with ${\mathbf{e}}\sim{\text{N}}\left( {0,{\mathbf{I}}\upsigma_{e}^{2} } \right)$.

Models (2), (3) and (4) are labeled as “REML-PED”, “REML-GRM” and “REML-PEDGRM”, respectively. Analyses using pedigree relationships were implemented in the DMU software [31]. The vectors ${\mathbf{g}}_{1}$ and ${\mathbf{g}}_{2}$ contain random effects with variance ${\text{var}}\left( {\mathbf{g}} \right) = {\mathbf{G}}\sigma_{g}^{2}$, where ${\mathbf{G}}$ is the GRM calculated following VanRaden’s method 1 [21]:

$${\mathbf{G}} = \frac{{\left( {{\mathbf{X}} - 2{\mathbf{p}}{\mathbf{1}}^{\prime } } \right)\left( {{\mathbf{X}} - 2{\mathbf{p}}\text{1}^{\prime } } \right)}}{{2\mathop \sum \nolimits_{j = 1}^{n} p_{j} \left( {1 - p_{j} } \right)}},$$

where ${\mathbf{X}}$ is the allele sharing matrix with the number of copies of the second allele, ${\mathbf{p}}$ is a vector with allele frequencies, and **1** is a vector of 1s. The factor $2\sum\nolimits_{j = 1}^{n} {p_{j} \left( {1 - p_{j} } \right)}$ scales ${\mathbf{G}}$ to be comparable to the pedigree relationship matrix. Analyses using the 50 k data GRM were implemented using the REML-GRM model of the GCTA software [32]. In addition, the REML-PEDGRM model was fitted with ${\mathbf{a}}_{2}$ and ${\mathbf{g}}_{2}$ simultaneously implemented in the DMU software. Reliabilities of DRP were not used in the models analyzed by DMU for consistency with the analyses using GCTA. The variance explained by pedigree relationship was re-scaled for REML-PED and REML-PEDGRM to use the same base genomic relationships, following Legarra [33].

## Results

### Contribution of different classes of genetic variants based on MAF to DRP variance

Additional file 1: Table S1 shows the proportion of DRP variance explained and standard error (SE) for variants partitioned into seven MAF groups for 17 traits and Additional file 2: Table S2 presents the same for variants partitioned into seven MAF groups and four LD groups for 17 traits. A substantial proportion of the variance estimates had large SE for most traits when variants were partitioned into seven MAF groups and four LD groups [see Additional file 2: Table S2]. A similar pattern of large SE for the estimates was observed when variants were partitioned into seven MAF groups and three LD groups. However, relatively better estimates were obtained when variants were partitioned into seven MAF groups only [see Additional file 1: Table S1]. Therefore, only results for variants partitioned into seven MAF groups are presented here. However, partitioning variants into seven MAF groups also resulted in several variance estimates with large SE, especially when the estimates were small [see Additional file 1: Table S1].

Interestingly, we observed that the relative contribution of variants with a MAF between 0.2 and 0.3 was substantially higher than that of other classes for MILK, FAT and PROT, as well as for LEG and LONG, while the imputed sequence variants were more or less evenly distributed across each MAF class (Fig. 1). This might be due to the *DGAT1* gene [34] (located on chromosome 14, position 1,802,265 bp with a MAF = 0.29), which is the largest milk-related QTL, explaining 11.2% of the DRP variance in MILK, 16.9% of the DRP variance in FAT and 2.9% of the DRP variance in PROT.

The proportion of DRP variance explained by rare (MAF < 0.01), low-frequency (MAF = 0.01–0.05) and common variants (i.e. MAF = 0.05–0.1, 0.1–0.2, 0.2–0.3, 0.3–0.4 and 0.4–0.5) in Additional file 1: Table S1 was divided by the total proportion of DRP variance explained and the results are summarized in Table 2 with three classes of variants (i.e. rare, low-frequency and common variants). The proportion of the DRP variance explained by the imputed sequence variants ranged from 0.406 (SE = 0.026) for TEMP to 0.872 (SE = 0.011) for MILK. The highest relative contribution among different classes of MAF was observed for the group of common variants (MAF ≥ 0.05) and ranged from 0.755 for HEALTH to 0.980 for BIRTH. For rare variants (MAF < 0.01), the contribution to the DRP variance explained was relatively small (ranging from 0 (with high SE) for FERT and BIRTH to 0.125 for HEALTH) compared with that from common variants (Table 2). The rare and low frequency variants (MAF < 0.05) contributed higher proportions of the explained DRP variance (in total >0.13 based on Table 2) for the health-related traits [i.e. fertility, other-diseases (health), longevity, feet and legs] compared with the production traits (in total <0.11 based on Table 2, i.e. yield index, protein yield and milk yield) (Table 2; Fig. 1).

**Table 2:** Relative contribution to the proportion of DRP variance explained by variants in different MAF classes for 17 traits

Traits and scenarios	Relative contribution of MAF classes to the explained DRP variance			Total proportion of DRP variance explained
	0.001–0.01	0.01–0.05	0.05–0.5	
YIELD	0.063	0.038	0.899	0.860
MILK	0.015	0.030	0.955	0.872
PROT	0.054	0.038	0.908	0.858
FAT	0.022	0.012	0.966	0.854
MILKORG	0.072	0.003	0.925	0.679
MILKSP	0.005	0.035	0.960	0.719
LONG	0.081	0.141	0.778	0.630
MASTI	0.019	0.000	0.981	0.669
HEALTH	0.125	0.121	0.755	0.514
LEG	0.059	0.149	0.796	0.525
CALV	0.037	0.000	0.963	0.507
BIRTH	0.000	0.020	0.980	0.602
FERT	0.000	0.133	0.867	0.600
BODY	0.088	0.026	0.886	0.568
GROWTH	0.010	0.087	0.903	0.814
TEMP	0.054	0.059	0.887	0.406
NTM	0.031	0.030	0.940	0.847

All the variants were partitioned into seven MAF classes. In this table, we report the proportion of DRP variance explained for three groups of MAF classes (rare: MAF < 0.01, low-frequency: 0.01 ≤ MAF < 0.05 and common: MAF ≥ 0.05). For the group of common variants, the proportion of DRP variance explained was equal to the sum of the proportions of DRP variance explained for classes of variants with MAF: 0.05–0.1; 0.1–0.2; 0.2–0.3; 0.3–0.4 and 0.4–0.5

### Proportion of DRP variance captured by pedigree and 50 k SNPs

The proportions of DRP variance explained for 17 traits by the different models (i.e. GREML-MS, REML-PED, REML-GRM and REML-PEDGRM) and using different information sources to construct relationship matrices (i.e. imputed sequence variants, 50 k SNPs or pedigree data) are in Table 3. Estimates of residual variance over the total variance of DRP are in Additional file 3: Table S3 and the Akaike information criterion (AIC) [35] of the different models are in Additional file 4: Table S4. We observed that estimates of the residual variance and total DRP variance were similar across all models and information sources for a given trait. Therefore, the proportion of DRP variance explained was comparable across models and data sources for a trait [see Additional file 3: Table S3]. For most traits, REML-PEDGRM had the lowest AIC value, which means that this model fit the data best, whereas for some traits, GREML-MS fit the data best [see Additional file 4: Table S4].

**Table 3:** Proportion of DRP variance explained using different methods

Traits	GREML-MS	REML-GRM	REML-PED	REML-PEDGRM
YIELD	0.860	0.845	0.923	0.941
MILK	0.872	0.844	0.887	0.927
PROT	0.858	0.847	0.943	0.963
FAT	0.854	0.840	0.898	0.914
MILKORG	0.679	0.703	0.811	0.816
MILKSP	0.719	0.715	0.748	0.840
LONG	0.630	0.606	0.884	0.881
MASTI	0.669	0.684	0.704	0.769
HEALTH	0.514	0.502	0.893	0.892
LEG	0.525	0.525	0.709	0.669
CALV	0.507	0.504	0.698	0.689
BIRTH	0.602	0.612	0.698	0.695
FERT	0.600	0.594	0.851	0.769
BODY	0.568	0.560	0.633	0.594
GROWTH	0.814	0.800	0.916	0.943
TEMP	0.406	0.403	0.645	0.645
NTM	0.847	0.839	–^a^	–

GREML-MS refers to estimation using the GREML-MS method with imputed sequence variants partitioned into MAF classes. REML-GRM refers to estimation using 50 k SNPs with the REML-GRM model implemented in GCTA. REML-PED refers to using pedigree relationship in the REML-PED model implemented in DMU. REML-PEDGRM refers to fitting both 50 k SNPs and pedigree relationship in the REML-PEDGRM model implemented in DMU

^a^The model did not converge

Imputed sequence variants explained more DRP variance than 50 k SNPs for most traits (Table 3). However, the DRP variance explained by imputed sequence variants was still smaller than the genetic variance estimated by using the pedigree-based relationship matrix; the difference was smallest for MILK (0.015) and largest for HEALTH (0.379).

The variance explained by fitting both pedigree and genomic relationship matrices (GRM) using the 50 k data in the PED + 50 k-DMU model, relative to the variance explained by the pedigree-based relationship matrix alone (PED-DMU), ranged from 109.2% for MASTI to 90.3% for FERT (Table 3). Furthermore, the proportion of explained DRP variance by 50 k-based GRM in the total explained genetic variance from both 50 k-based GRM and pedigree-based relationship matrix using PED + 50 k-DMU model ranged from 79.8% for FAT to 26.1% for HEALTH. These results indicate that common variants were able to capture a large proportion of the genetic variance, especially for production traits.

## Discussion

### Contribution of MAF classes to the variance of DRP

We estimated the relative contribution of genetic variants in different MAF classes to the explained DRP variance. However, many of these estimates had large SE when variants were partitioned into MAF and LD groups, or only into MAF groups. Although the method of partitioning variants in different MAF and LD groups was used to estimate heritability accurately in human data, many of our estimates for this bovine population had large SE. The number of individuals used in the human study was 44,126 [18], which was much larger than the sample size used in this study in cattle (~5000). Therefore, to obtain reliable estimates of the explained DRP variance for different MAF classes, a larger sample size is needed in cattle population.

For all traits, the relative contribution of rare and low frequency variants to the proportion of DRP variance explained was small compared to the contribution of common variants. For health-related traits, the proportion of DRP variance explained by rare and low frequency variants was on average more than 13%, which was high compared to that for production traits. Gonzalez-Recio et al. [36] also reported that rare variants explained 14% of the genetic variance for fertility in Holstein cattle. These results reflect that the genetic architecture of health-related traits probably differs from that of production traits in the sense that rare variants have a relatively larger impact on variation in health-related traits. This is expected since selection is purging the rare variants with a negative effect on fitness, for example, the rare deleterious variants will be purged by selection. However, the rare and low-frequency variants with a positive effect such as selective advantage could be very relevant for long-term selection response if they have a medium to large effect [37].

The variance explained by the class of variants with a MAF between 0.2 and 0.3 was low (0.001) for HEALTH (Fig. 1) and [see Additional file 1: Table S1] but is probably not biologically relevant given the large SE of this estimate. When we compared DRP variance among the traits analysed, we observed no specific pattern of rare frequency variants explaining more DRP variance than low-frequency variants. However, again the large SE for the estimates may mask any pattern that may be present. For YIELD, PROT, MILKORG, MASTI, CALV and BODY, rare variants explained more variance than low-frequency variants; for MILK, FAT, MILKSP, LEG, BIRTH, FERT and GROWTH, low-frequency variants explained more variance than rare variants; and for HEALTH, TEMP and NTM, rare variants explained a similar proportion of variance as that found for low-frequency variants. Rare or low-frequency variants with more explained DRP variance for different traits might reflect the genetic architecture (i.e. what kind of causal variants underlie the traits). Rare or low-frequency causal variants generally have larger effect sizes [38] and might also have a larger contribution to phenotypic variation. For human height, rare variants explained 8.4% of the genetic variance and variants with a MAF ranging from 0.01 to 0.1 explained 13% of the genetic variance [18]. However, a previous study on bovine fertility reported that rare variants explained 14% of the genetic variance, while low-frequency variants (0.01 < MAF ≤ 0.05) explained 0% of the genetic variance [36], but this may result from an imprecise estimate due to a small sample size, as in our study.

Computing correlations between the GRM that was constructed with rare variants and with the GRM constructed with other MAF class variants suggested that the GRM that were constructed with common variants captured at least some of the variance that was captured by the GRM built with rare variants (Table 4). Table 5 shows the comparison between expected and estimated variance explained by each MAF class for LEG. The differences between estimated and expected variances for the rare and low-frequency variants for LEG were large (0.137 and −0.125 for expected variances using VanRaden’s methods 1 and 2, respectively) and the estimated variance was actually intermediate to the expected variances obtained with the two VanRaden methods [21]. The difference between expected variances with the two VanRaden methods was much larger for rare and low-frequency variants than for common variants. Thus, it might be necessary to correct the current model (two VanRaden’s methods), as proposed by Speed et al. [39]; generally, the genomic relationship matrix (${\mathbf{X}}_{i,j} )$ is calculated as:

$${\mathbf{X}}_{i,j} = \left( {{\mathbf{S}}_{i,j} - 2f_{j} } \right) \times \left( {2f_{j} \left( {1 - f_{j} } \right)} \right)^{\alpha /2} ,$$

where ${\mathbf{S}}_{i,j}$ is the number of copies of the minor allele carried by individual $i$ at SNP $j$, $f_{j}$ is the allele frequency at the SNP $j$ and $\alpha$ is commonly set to −1 in human genetics and to 0 in animal and plant genetics [39]. Speed et al. [39] found that the optimal $\alpha$ was −0.25 for their human data. [39]. Our results support the need of exploring the optimal $\alpha$ to be used for constructing genomic relationship matrices.

**Table 4:** Correlations of the off-diagonal elements of the genomic relationship matrix (GRM) built using variants in different classes of MAF

MAF class of variants used to construct the GRM	0.001–0.01	0.01–0.05	0.05–0.1	0.1–0.2	0.2–0.3	0.3–0.4	0.4–0.5
0.001–0.01	1.000	0.546	0.372	0.339	0.322	0.313	0.310
0.01–0.05		1.000	0.811	0.756	0.723	0.704	0.696
0.05–0.1			1.000	0.911	0.865	0.845	0.835
0.1–0.2				1.000	0.948	0.925	0.915
0.2–0.3					1.000	0.962	0.950
0.3–0.4						1.000	0.968
0.4–0.5							1.000

**Table 5:** Expectations and estimates of the proportion of variance explained by the variants in different MAF classes using imputed sequence data for the feet and legs trait

	MAF class						
	0.001–0.01	0.01–0.05	0.05–0.1	0.1–0.2	0.2–0.3	0.3–0.4	0.4–0.5
Expectation VR1^a^	0.006	0.065	0.079	0.252	0.210	0.194	0.193
Expectation VR2^a^	0.147	0.186	0.134	0.169	0.132	0.118	0.114
Estimate^b^	0.059	0.149	0.025	0.053	0.299	0.417	0.002

All proportions are scaled to sum to 1 across all MAF classes

^a^Expectations of the proportion of variance explained based on the assumption of VanRaden’s methods 1 (VR1) and 2 (VR2); see [21]

^b^Estimated proportion of DRP variance explained for feet and legs using the GREML-MS method with partitioning of imputed sequence variants into seven MAF groups

It was previously shown that the contribution of rare variants to phenotypic variance of disease and stature in humans is large [18, 40]. In dairy cattle, we observed that rare variants play a bigger role for health-related traits than for production traits. Similar to the findings for human height, we also observed that rare variants contributed significantly (the contribution of rare variants for BODY was 0.088) to the body conformation index, for which stature is the main component trait.

In our study, the sequence data that was used to estimate the variance explained by different MAF classes of variants was imputed sequence data. Imputation errors can result in underestimation of the variance explained by rare variants since they typically have a lower imputation accuracy [16]. The average imputation accuracy for rare variants in this study was 0.85, compared to 0.92 for other variants [see Additional file 1: Table S1], which indicates that imputation accuracy may be an important contributor in our study. The 17 traits studied in this analysis are all highly polygenic traits that are affected by a large number of loci. To better study rare variants, next-generation sequencing data from considerably more individuals in the reference population may be useful to improve imputation accuracy and reduce the cut-off threshold for MAF. In addition, the number of animals with phenotypes should be increased to obtain more reliable variance component estimates.

The models used in this study were originally developed to account for LD structure in human data. The LD structure observed from genome-wide loci in cattle differs greatly from that in humans, in that LD persists across much longer ranges and the LD scores are much higher in cattle than in humans, see [18] and Additional file 5: Figure S1; i.e. the LD score was in most cases higher than 1000 in cattle, while in humans it is lower than 200. Due to close family structures in cattle and the resulting LD structure, correlations between the GRM-matrices based on different MAF classes may be higher in bovine than in human data. Figure 1a in Lee et al. [41] shows that the estimated variances were very similar for each human chromosome, regardless of whether all chromosomes were fitted simultaneously or separately. Conversely, Daetwyler et al. [42] showed that SNPs from a single chromosome can achieve up to 86% of the accuracy for genomic predictions using all (50 k) SNPs. Strong LD and resulting high correlations between effects is probably the main reason why the data did not contain enough information for the model to accurately partition variances by MAF class. Thus, when we partitioned the variants into LD groups, the SE for the estimates of DRP variance explained within each MAF class were large. We showed that the correlations between GRM that were built with common variants were high (more than 0.6), while correlations between GRM that were built with rare variants and common variants were low (ranging from 0.3 to 0.4) (Table 4). Therefore, for bovine data, due to the strong LD, the variance explained by a certain MAF class of common variants can also be explained by another class of common variants, but probably less by rare variants.

### Proportion of DRP variance captured by pedigree and 50 k SNPs

We estimated the proportion of variance in DRP explained for 17 traits using different models and different data sources (Table 3). Imputed sequence variants explained a higher proportion of the DRP variance than the 50 k SNPs for most traits. However, the increase in variance explained was small (Table 3).

For all traits, estimation of DRP variance based on pedigree data explained the largest contribution of the total variance of DRP. This result is in line with other studies that used 50 k SNPs to construct the GRM [7, 8, 11]. The DRP were on progeny test bulls with adjustment for non-genetic effects with a pedigree-based model. Because the estimation and deregression process was based on a pedigree-based model, it is not surprising that the pedigree-based model explained the largest proportion of variance in DRP. In fact, the REML-PED model is expected to yield EBV that are very similar to the EBV that were used to compute the DRP [43]. For most health-related traits, the proportion of DRP variance estimated from pedigree relationships was small because the reliabilities of EBV for these traits were low.

## Conclusions

Our results show that the 50 k SNP chip can explain most of the genetic variance estimated by using pedigree relationships and even that estimated by using whole-genome sequence. We observed that using high-density SNPs resulted in only a limited increase in the DRP variance explained. As a result, it is necessary to include pedigree information, i.e. polygenic effects, in genomic prediction in dairy cattle to capture variance that is not captured by genomic markers. Our study also showed the relative importance of rare and low-frequency genomic variants for 17 traits in dairy cattle. Although a human study showed that partitioning variants in different MAF and LD groups decreased the bias of heritability estimates, many of our estimates for the bovine population had high SE. To obtain a reliable estimate of the explained DRP variance for different MAF classes, a larger sample size is needed.

## Additional files

**Additional file 1: Table S1.** Number of variants and imputation accuracy for each MAF class and proportion of DRP variance explained and standard errors for seven MAF classes without partitioning variants into LD groups for 17 traits. The number of variants for each MAF class was presented as the number of variants ± standard error. The imputation accuracy was reported using INFO values from *MINIMAC2* imputation. The imputation accuracies were presented as mean imputation accuracy ± standard deviation. The numbers were also presented as the proportion of explained DRP variance ± standard error. For each column of the table, the imputed sequence variants were classified into seven classes based on their MAF (0.001–0.01; 0.01–0.05; 0.05–0.1; 0.1–0.2; 0.2–0.3; 0.3–0.4 and 0.4–0.5). “-” means that there is no result for this case. Estimates that are larger than one time the standard error are in boldface.

**Additional file 2: Table S2.** Number of variants in different MAF classes, imputation accuracy for different MAF classes and proportion of DRP variance explained and standard errors for seven MAF by four LD classes for 17 traits. The numbers were presented as the proportion of explained DRP variance ± standard error. The traits where the model did not converge were not presented in this table. For each column of the table, the imputed sequence variants were classified into 7 classes based on their MAF (0.001–0.01; 0.01–0.05; 0.05–0.1; 0.1–0.2; 0.2–0.3; 0.3–0.4 and 0.4–0.5). For each row of the table, the variants within each of the 7 MAF classes were stratified into 4 equally sized LD groups based on increasing mean LD scores, resulting in 28 groups in total. Estimates that are larger than one time the standard error are in boldface.

**Additional file 3: Table S3.** Estimates of residual and total variance of DRP using different models and different information sources to construct the GRM. The total explained DRP variance for PROT in PED-DMU is scaled to 100.0 and used as a reference to scale other numbers across models and traits. There are two rows for each trait. The relative residual variance is presented on the first row and total variance of DRP is shown on the second row. “GREML-MS” is the relative residual variance and DRP variance calculated using the GREML-MS method with partitioning of imputed sequence variants into MAF groups. “REML-GRM” is the relative residual variance and DRP variance calculated by fitting 50 k SNPs with the REML-GRM model implemented in GCTA. “REML-PED” is the relative residual variance and DRP variance calculated by fitting pedigree relationships with the REML-PED model implemented in DMU. “REML-PEDGRM” is the relative residual variance and DRP variance calculated by fitting both 50 k SNPs and pedigree relationships with the REML-PEDGRM model implemented in DMU. “-” means that the model did not converge.

**Additional file 4: Table S4.** The Akaike information criterion (AIC) for different models compared to REML-PED. The log likelihoods for different models were used to calculate AIC following [35]. The AIC using “REML-PED” was scaled to zero for each trait and the AIC for the other models were expressed as the difference from AIC in “REML-PED”. GREML-MS” is the relative residual variance and DRP variance calculated using the GREML-MS method with partitioning of imputed sequence variants into MAF groups. “REML-GRM” is the relative residual variance and DRP variance calculated by fitting 50 k SNPs with the REML-GRM model implemented in GCTA. “REML-PED” is the relative residual variance and DRP variance calculated by fitting pedigree relationships with the REML-PED model implemented in DMU. “REML-PEDGRM” is the relative residual variance and DRP variance calculated by fitting both 50 k SNPs and pedigree relationships with the REML-PEDGRM model implemented in DMU. Results for the model that did not converge for the trait are not presented.

**Additional file 5: Figure S1.** LD score on a segment of chromosome 1 in a sample of Holstein individuals. The yellow dots are the LD score for each variant. The LD score was defined as the sum of the LD measure r^2^ between this SNP and other SNPs in a 20-Mb region centered on this SNP. The blue dots are the average LD score for a sliding window of 100 kb.
