# Supplementary material for: Contribution of rare and low-frequency whole-genome sequence variants to complex traits variation in dairy cattle
Source: Genet Sel Evol. 2017 Aug 1;49:60. doi: 10.1186/s12711-017-0336-z (PMC5539983; doi:10.1186/s12711-017-0336-z)
Supplement: Supplementary file 1 — Additional file 1: Table S1. Number of variants and imputation accuracy for each MAF class and proportion of DRP variance explained and standard errors for seven MAF classes without partitioning variants into LD groups for 17 traits. The number of variants for each MAF class was presented as the number of variants ± standard error. The imputation accuracy was reported using INFO values from MINIMAC2 imputation. The imputation accuracies were presented as mean imputation accuracy ± standard deviation. The numbers were also presented as the proportion of explained DRP variance ± standard error. For each column of the table, the imputed sequence variants were classified into seven classes based on their MAF (0.001–0.01; 0.01–0.05; 0.05–0.1; 0.1–0.2; 0.2–0.3; 0.3–0.4 and 0.4–0.5). “-” means that there is no result for this case. Estimates that are larger than one time the standard error are in boldface. [file 12711_2017_336_MOESM1_ESM.docx]

Table S1 Number of variants and imputation accuracy for each MAF class and proportion of DRP variance explained and standard errors for seven MAF classes without partitioning variants into LD groups for 17 traits

| Traits\MAF | 0.001-0.01 | 0.01-0.05 | 0.05-0.1 | 0.1-0.2 | 0.2-0.3 | 0.3-0.4 | 0.4-0.5 | Total explained DRP variance |
| --- | --- | --- | --- | --- | --- | --- | --- | --- |
| Number of variants | 2,910,287 | 3,679,012 | 2,649,046 | 3,336,965 | 2,605,208 | 2,327,656 | 2,247,658 | 19,755,832 |
| Imputation accuracy | 0.850±0.233 | 0.873±0.215 | 0.892±0.188 | 0.916±0.162 | 0.936±0.141 | 0.945±0.132 | 0.948±0.132 | 0.888±0.216 |
| YIELD | **0.054±0.017** | **0.033±0.029** | **0.133±0.040** | **0.058±0.051** | **0.222±0.060** | **0.105±0.058** | **0.256****±0.054** | **0.860±0.013** |
| MILK | **0.013±0.013** | **0.026±0.024** | **0.090±0.035** | 0.000±0.046 | **0.366±0.056** | **0.095±0.055** | **0.282±0.051** | **0.872±0.011** |
| PROT | **0.046±0.015** | **0.033±0.027** | **0.138±0.038** | 0.005±0.048 | **0.219±0.058** | **0.129±0.057** | **0.286±0.053** | **0.858±0.012** |
| FAT | **0.019±0.013** | 0.010±0.024 | **0.041±0.034** | 0.000±0.046 | **0.363±0.058** | **0.139±0.056** | **0.282±0.051** | **0.854±0.012** |
| MILKORG | **0.049±0.037** | 0.002±0.058 | 0.034±0.069 | 0.053±0.098 | 0.088±0.108 | **0.416±0.115** | 0.036±0.092 | **0.679±0.035** |
| MILKSP | 0.004±0.015 | 0.025±0.030 | **0.084±0.040** | **0.168±0.056** | **0.064±0.062** | **0.307±0.064** | **0.068±0.054** | **0.719±0.018** |
| LONG | **0.051±0.021** | **0.089±0.035** | 0.036±0.042 | **0.072±0.059** | **0.201±0.068** | **0.137±0.066** | 0.044±0.057 | **0.630±0.022** |
| MASTI | 0.013±0.016 | 0.000±0.029 | 0.013±0.038 | **0.156±0.057** | **0.197±0.062** | **0.121±0.063** | **0.168±0.055** | **0.669±0.020** |
| HEALTH | **0.064±****0.023** | **0.062±0.036** | **0.083±0.044** | **0.081±0.057** | 0.001±0.061 | **0.159±0.064** | **0.065±0.056** | **0.514±0.024** |
| LEG | **0.031±0.020** | **0.078±0.033** | 0.013±0.039 | 0.028±0.055 | **0.157±0.063** | **0.219±0.063** | 0.000±0.051 | **0.525±0.024** |
| CALV | 0.019±0.019 | 0.000±0.033 | **0.106±0.043** | **0.089±0.056** | **0.187±0.063** | **0.106±0.061** | 0.000±0.053 | **0.507±0.024** |
| BIRTH | 0.000±0.017 | 0.012±0.031 | **0.078±0.043** | **0.215±0.059** | **0.098±0.062** | **0.157±0.064** | 0.043±0.055 | **0.602±0.023** |
| FERT | 0.000±0.018 | **0.080±0.034** | **0.085±0.042** | **0.064±0.056** | **0.140±0.064** | **0.123±0.063** | **0.107±0.055** | **0.600±0.021** |
| BODY | **0.050±0.020** | 0.015±0.032 | **0.045±0.040** | **0.129±0.058** | **0.134±0.064** | 0.057±0.061 | **0.138±0.058** | **0.568±0.022** |
| GROWTH | 0.008±0.015 | **0.071±0.029** | 0.000±0.040 | **0.167±0.058** | **0.233±0.064** | **0.148±0.065** | **0.188±0.057** | **0.814±0.016** |
| TEMP | **0.022±0.021** | 0.024±0.034 | 0.026±0.042 | **0.105±0.059** | **0.184±0.065** | 0.000±0.063 | 0.045±0.053 | **0.406±0.026** |
| NTM | **0.026±0.015** | 0.025±0.028 | **0.073±0.039** | **0.163±0.054** | **0.321±0.059** | 0.079±0.058 | **0.161±0.052** | **0.847±0.012** |
