# Supplementary material for: Contribution of rare and low-frequency whole-genome sequence variants to complex traits variation in dairy cattle
Source: Genet Sel Evol. 2017 Aug 1;49:60. doi: 10.1186/s12711-017-0336-z (PMC5539983; doi:10.1186/s12711-017-0336-z)
Supplement: Supplementary file 2 — Additional file 2: Table S2. Number of variants in different MAF classes, imputation accuracy for different MAF classes and proportion of DRP variance explained and standard errors for seven MAF by four LD classes for 17 traits. The numbers were presented as the proportion of explained DRP variance ± standard error. The traits where the model did not converge were not presented in this table. For each column of the table, the imputed sequence variants were classified into 7 classes based on their MAF (0.001–0.01; 0.01–0.05; 0.05–0.1; 0.1–0.2; 0.2–0.3; 0.3–0.4 and 0.4–0.5). For each row of the table, the variants within each of the 7 MAF classes were stratified into 4 equally sized LD groups based on increasing mean LD scores, resulting in 28 groups in total. Estimates that are larger than one time the standard error are in boldface. [file 12711_2017_336_MOESM2_ESM.docx]

Table S2 Number of variants in different MAF classes, imputation accuracy for different MAF classes and proportion of DRP variance explained and standard errors for seven MAF by four LD classes for 17 traits

| Traits | MAF/LD groups | 0.001-0.01 | 0.01-0.05 | 0.05-0.1 | 0.1-0.2 | 0.2-0.3 | 0.3-0.4 | 0.4-0.5 | Total explained DRP variance |
| --- | --- | --- | --- | --- | --- | --- | --- | --- | --- |
| YIELD | 1 | **0.018± 0.016** | 0.015± 0.021 | **0.033± 0.025** | 0.016± 0.029 | 0.050± 0.032 | 0.001± 0.030 | **0.093± 0.028** | **0.868±0.012** |
|  | 2 | 0.009± 0.019 | 0.003± 0.025 | **0.062± 0.030** | 0.017± 0.034 | **0.085± 0.036** | 0.002± 0.034 | 0.024± 0.028 |  |
|  | 3 | 0.015± 0.018 | **0.025± 0.025** | 0.001± 0.027 | 0.001± 0.036 | **0.096± 0.037** | **0.076± 0.035** | **0.083± 0.032** |  |
|  | 4 | 0.001± 0.009 | 0.001± 0.017 | **0.042± 0.021** | **0.048± 0.027** | 0.001± 0.026 | 0.016± 0.028 | **0.037± 0.025** |  |
| PROT | 1 | **0.024±0.015** | 0.013±0.021 | **0.030±0.024** | 0.001±0.028 | **0.061±0.032** | 0.004±0.030 | **0.120±0.029** | **0.862±0.012** |
|  | 2 | 0.002±0.017 | 0.017±0.024 | **0.071±0.029** | 0.006±0.032 | **0.082±0.035** | **0.051±0.033** | 0.002±0.027 |  |
|  | 3 | 0.005±0.016 | 0.001±0.022 | 0.001±0.025 | 0.001±0.034 | **0.073±0.034** | **0.044±0.033** | **0.112±0.031** |  |
|  | 4 | 0.003±0.008 | 0.001±0.014 | **0.048±0.021** | **0.031±0.024** | 0.001±0.025 | **0.027±0.027** | **0.033±0.024** |  |
| FAT | 1 | **0.014±0.014** | 0.001±0.020 | 0.006±0.023 | 0.007±0.029 | 0.009±0.031 | **0.096±0.036** | **0.145±0.031** | **0.853±0.012** |
|  | 2 | 0.003±0.017 | 0.016±0.023 | 0.025±0.027 | 0.001±0.033 | **0.288±0.041** | 0.001±0.035 | 0.001±0.028 |  |
|  | 3 | 0.011±0.016 | 0.015±0.023 | 0.001±0.023 | 0.006±0.031 | 0.026±0.031 | **0.067±0.031** | **0.053±0.028** |  |
|  | 4 | 0.001±0.011 | 0.001±0.015 | **0.016±0.016** | 0.014±0.020 | 0.001±0.022 | 0.010±0.023 | **0.024±0.021** |  |
| MILKORG | 1 | 0.001±0.017 | 0.001±0.025 | 0.001±0.028 | **0.109±0.037** | 0.017±0.035 | **0.092±0.037** | 0.001±0.029 | **0.735±0.018** |
|  | 2 | 0.001±0.022 | **0.033±0.029** | **0.032±0.030** | 0.001±0.038 | **0.084±0.040** | **0.072±0.037** | 0.023±0.031 |  |
|  | 3 | 0.026±0.020 | 0.008±0.025 | 0.003±0.027 | 0.003±0.036 | **0.057±0.036** | **0.057±0.037** | 0.029±0.031 |  |
|  | 4 | 0.001±0.014 | 0.001±0.017 | 0.011±0.018 | 0.001±0.024 | **0.033±0.027** | **0.038±0.028** | 0.011±0.023 |  |
| MILKSP | 1 | 0.011±0.019 | 0.001±0.025 | 0.001±0.028 | **0.100±0.038** | 0.017±0.036 | **0.107±0.037** | 0.001±0.031 | **0.722±0.019** |
|  | 2 | 0.001±0.023 | 0.017±0.029 | **0.046±0.031** | 0.002±0.037 | **0.047±0.039** | 0.006±0.037 | **0.063±0.034** |  |
|  | 3 | 0.001±0.024 | **0.029±0.028** | 0.001±0.028 | 0.004±0.034 | 0.005±0.036 | **0.113±0.038** | **0.043±0.033** |  |
|  | 4 | 0.013±0.018 | 0.001±0.019 | **0.019±0.018** | **0.038±0.024** | 0.001±0.025 | **0.040±0.028** | 0.001±0.022 |  |
| LONG | 1 | **0.034±0.024** | 0.001±0.030 | **0.053±0.033** | 0.001±0.038 | **0.112±0.041** | 0.001±0.037 | 0.008±0.031 | **0.643±0.022** |
|  | 2 | 0.001±0.028 | **0.064±0.035** | 0.001±0.033 | 0.025±0.041 | **0.055±0.042** | **0.071±0.041** | 0.001±0.032 |  |
|  | 3 | 0.004±0.026 | 0.008±0.029 | 0.003±0.030 | 0.001±0.038 | 0.006±0.037 | 0.022±0.037 | **0.047±0.034** |  |
|  | 4 | **0.018±0.018** | 0.003±0.022 | 0.001±0.020 | **0.036±0.031** | **0.037±0.031** | **0.034±0.030** | 0.001±0.027 |  |
| MASTI | 1 | 0.001±0.020 | 0.001±0.026 | 0.019±0.029 | 0.012±0.034 | 0.026±0.034 | **0.033±0.033** | 0.020±0.026 | **0.683±0.021** |
|  | 2 | 0.001±0.025 | **0.074±0.034** | 0.001±0.032 | **0.073±0.041** | **0.110±0.041** | 0.013±0.038 | **0.054±0.034** |  |
|  | 3 | **0.034±0.026** | 0.001±0.030 | 0.002±0.028 | **0.054±0.039** | **0.048±0.038** | 0.005±0.035 | 0.002±0.031 |  |
|  | 4 | 0.010±0.017 | 0.004±0.020 | 0.005±0.018 | 0.001±0.024 | 0.005±0.028 | **0.038±0.030** | **0.043±0.027** |  |
| HEALTH | 1 | 0.023± 0.025 | 0.024± 0.030 | **0.034± 0.032** | 0.024± 0.035 | 0.008± 0.034 | **0.038± 0.035** | 0.010± 0.029 | **0.527±0.024** |
|  | 2 | 0.002± 0.028 | 0.019± 0.035 | **0.038± 0.035** | 0.001± 0.042 | 0.001± 0.040 | **0.052± 0.039** | 0.001± 0.032 |  |
|  | 3 | 0.026± 0.028 | 0.001± 0.032 | 0.001± 0.032 | **0.093± 0.041** | 0.001± 0.039 | **0.040± 0.039** | 0.016± 0.033 |  |
|  | 4 | 0.001± 0.016 | 0.014± 0.022 | 0.014± 0.023 | 0.001± 0.026 | 0.001± 0.028 | 0.008± 0.031 | **0.043± 0.028** |  |
| CALV | 1 | 0.010± 0.023 | 0.001± 0.027 | **0.032± 0.032** | 0.012± 0.036 | **0.071± 0.037** | 0.001± 0.034 | 0.019± 0.031 | **0.525±0.025** |
|  | 2 | 0.001± 0.027 | 0.001± 0.032 | 0.007± 0.033 | 0.023± 0.040 | **0.041± 0.041** | **0.072± 0.041** | 0.001± 0.034 |  |
|  | 3 | **0.033 ± 0.028** | 0.029± 0.033 | 0.020± 0.031 | 0.001± 0.038 | 0.014± 0.038 | **0.076± 0.040** | 0.001± 0.035 |  |
|  | 4 | 0.001± 0.018 | 0.001± 0.022 | **0.022± 0.021** | 0.015± 0.026 | 0.012± 0.027 | 0.001± 0.026 | 0.017± 0.025 |  |
| BIRTH | 1 | **0.047± 0.023** | 0.001± 0.027 | 0.012± 0.031 | 0.018± 0.035 | 0.001± 0.035 | **0.038± 0.036** | **0.077± 0.034** | **0.636±0.023** |
|  | 2 | 0.001± 0.026 | **0.042± 0.034** | 0.001± 0.032 | **0.085± 0.041** | 0.010± 0.038 | 0.001± 0.037 | 0.001± 0.031 |  |
|  | 3 | 0.001± 0.025 | 0.001± 0.029 | **0.092± 0.034** | 0.001± 0.040 | 0.036± 0.038 | **0.074± 0.037** | 0.003± 0.032 |  |
|  | 4 | 0.001± 0.018 | 0.006± 0.020 | 0.001± 0.021 | **0.069± 0.030** | 0.009± 0.029 | 0.016± 0.030 | 0.001± 0.025 |  |
| FERT | 1 | **0.038 ± 0.023** | 0.001± 0.028 | **0.043± 0.030** | 0.020± 0.035 | **0.040± 0.034** | 0.021± 0.035 | 0.014± 0.027 | **0.607±0.022** |
|  | 2 | 0.001± 0.025 | **0.042± 0.033** | 0.001± 0.033 | **0.045± 0.041** | 0.024± 0.039 | **0.076± 0.040** | 0.006± 0.033 |  |
|  | 3 | 0.001± 0.024 | **0.036± 0.032** | **0.033± 0.031** | 0.001± 0.039 | 0.030± 0.037 | 0.007± 0.035 | **0.056± 0.034** |  |
|  | 4 | 0.001± 0.016 | 0.004± 0.019 | 0.002± 0.018 | 0.030± 0.025 | 0.002± 0.027 | **0.032± 0.028** | 0.008± 0.024 |  |
| GROWTH | 1 | **0.018± 0.017** | 0.002± 0.023 | 0.001± 0.028 | **0.130± 0.039** | 0.020± 0.036 | **0.063± 0.036** | **0.057± 0.032** | **0.819±0.016** |
|  | 2 | 0.013± 0.020 | 0.001± 0.027 | 0.010± 0.031 | 0.070± 0.039 | **0.046± 0.037** | 0.004± 0.036 | **0.058± 0.033** |  |
|  | 3 | 0.001± 0.019 | 0.012± 0.026 | 0.001± 0.030 | 0.016± 0.036 | 0.025± 0.038 | 0.029± 0.036 | **0.077± 0.033** |  |
|  | 4 | 0.001± 0.014 | **0.050± 0.022** | 0.001± 0.020 | 0.001± 0.026 | **0.096± 0.034** | 0.023± 0.029 | 0.001± 0.024 |  |
| TEMP | 1 | 0.015± 0.025 | 0.001± 0.031 | 0.016± 0.033 | **0.039± 0.039** | **0.054± 0.039** | 0.036± 0.037 | 0.001± 0.032 | **0.429±0.027** |
|  | 2 | 0.001± 0.030 | 0.001± 0.035 | 0.001± 0.034 | 0.001± 0.042 | **0.080± 0.044** | 0.018± 0.039 | 0.004± 0.031 |  |
|  | 3 | 0.026± 0.030 | 0.003± 0.032 | **0.037± 0.034** | 0.010± 0.038 | 0.009± 0.037 | 0.001± 0.035 | 0.001± 0.032 |  |
|  | 4 | 0.001± 0.018 | 0.010± 0.020 | 0.006± 0.021 | 0.017± 0.027 | 0.022± 0.030 | 0.001± 0.030 | **0.027± 0.025** |  |
| NTM | 1 | 0.026± 0.017 | 0.001± 0.022 | **0.032± 0.026** | 0.019± 0.031 | **0.078± 0.033** | 0.028± 0.032 | **0.043± 0.028** | **0.848±0.013** |
|  | 2 | 0.001± 0.020 | 0.020± 0.026 | 0.003± 0.027 | 0.018± 0.035 | **0.131± 0.038** | **0.050± 0.036** | **0.049± 0.031** |  |
|  | 3 | **0.022± 0.020** | 0.003± 0.024 | 0.026± 0.027 | **0.044± 0.036** | **0.064± 0.035** | 0.014± 0.033 | 0.018± 0.029 |  |
|  | 4 | 0.004± 0.011 | 0.001± 0.017 | 0.008± 0.019 | **0.069± 0.028** | **0.037± 0.029** | 0.001± 0.027 | **0.042± 0.026** |  |
