# Supplementary material for: Contribution of rare and low-frequency whole-genome sequence variants to complex traits variation in dairy cattle
Source: Genet Sel Evol. 2017 Aug 1;49:60. doi: 10.1186/s12711-017-0336-z (PMC5539983; doi:10.1186/s12711-017-0336-z)
Supplement: Supplementary file 3 — Additional file 3: Table S3. Estimates of residual and total variance of DRP using different models and different information sources to construct the GRM. The total explained DRP variance for PROT in PED-DMU is scaled to 100.0 and used as a reference to scale other numbers across models and traits. There are two rows for each trait. The relative residual variance is presented on the first row and total variance of DRP is shown on the second row. “GREML-MS” is the relative residual variance and DRP variance calculated using the GREML-MS method with partitioning of imputed sequence variants into MAF groups. “REML-GRM” is the relative residual variance and DRP variance calculated by fitting 50 k SNPs with the REML-GRM model implemented in GCTA. “REML-PED” is the relative residual variance and DRP variance calculated by fitting pedigree relationships with the REML-PED model implemented in DMU. “REML-PEDGRM” is the relative residual variance and DRP variance calculated by fitting both 50 k SNPs and pedigree relationships with the REML-PEDGRM model implemented in DMU. “-” means that the model did not converge. [file 12711_2017_336_MOESM3_ESM.docx]

**Table S3 Estimates of residual and total variance of DRP using different models and different information sources to construct the GRM**

| Trait | Residual variance | | | |
| --- | --- | --- | --- | --- |
|  | Ttotal variance of DRP | | | |
|  | GREML-MS | REML-GRM | REML-PED | REML-PEDGRM |
| PROT | 14.1 | 15.9 | 1.6 | 6.0 |
|  | 99.4 | 104.0 | 100.0 | 98.0 |
| FERT | 78.7 | 82.0 | 23.9 | 42.0 |
|  | 196.6 | 202.1 | 211.7 | 199.0 |
| HEALTH | 113.7 | 122.4 | 18.0 | 37.4 |
|  | 233.8 | 245.6 | 266.3 | 248.4 |
| LONG | 62.2 | 74.7 | 20.8 | 28.0 |
|  | 181.6 | 189.9 | 190.8 | 182.4 |
| MILKSP | 61.6 | 64.1 | 14.9 | 33.6 |
|  | 219.6 | 225.2 | 221.2 | 224.6 |
| YIELD | 12.5 | 14.6 | 3.4 | 0.04 |
|  | 89.2 | 94.2 | 91.0 | 88.3 |
| CALV | 80.3 | 83.3 | 47.5 | 51.2 |
|  | 162.9 | 168.1 | 174.6 | 171.8 |
| MILK | 14.1 | 17.0 | 7.9 | 6.8 |
|  | 109.7 | 109.0 | 105.1 | 104.4 |
| BIRTH | 60.4 | 60.7 | 58.4 | 47.2 |
|  | 180.911 | 156.5 | 147.2 | 154.3 |
| FAT | 14.4 | 15.9 | 6.2 | 7.4 |
|  | 98.7 | 99.4 | 99.3 | 94.6 |
| GROWTH | 23.0 | 26.2 | 5.7 | 6.1 |
|  | 128.7 | 131.0 | 134.2 | 132.4 |
| LEG | 134.8 | 140.1 | 80.3 | 95.0 |
|  | 284.1 | 294.9 | 308.2 | 296.9 |
| MASTI | 33.4 | 32.8 | 27.0 | 22.6 |
|  | 100.9 | 103.7 | 101.6 | 101.4 |
| MILKORG | 50.9 | 49.8 | 26.6 | 28.4 |
|  | 158.7 | 167.8 | 171.3 | 163.9 |
| NTM | 14.4 | 15.9 | - | - |
|  | 94.3 | 99.0 | - | - |
| TEMP | 127.2 | 131.7 | 75.1 | 76.9 |
|  | 214.0 | 220.6 | 229.4 | 227.5 |
| BODY | 70.5 | 74.2 | 58.3 | 65.9 |
|  | 163.3 | 168.6 | 171.7 | 164.4 |
