# Supplementary material for: Contribution of rare and low-frequency whole-genome sequence variants to complex traits variation in dairy cattle
Source: Genet Sel Evol. 2017 Aug 1;49:60. doi: 10.1186/s12711-017-0336-z (PMC5539983; doi:10.1186/s12711-017-0336-z)
Supplement: Supplementary file 4 — Additional file 4: Table S4. The Akaike information criterion (AIC) for different models compared to REML-PED. The log likelihoods for different models were used to calculate AIC following [35]. The AIC using “REML-PED” was scaled to zero for each trait and the AIC for the other models were expressed as the difference from AIC in “REML-PED”. GREML-MS” is the relative residual variance and DRP variance calculated using the GREML-MS method with partitioning of imputed sequence variants into MAF groups. “REML-GRM” is the relative residual variance and DRP variance calculated by fitting 50 k SNPs with the REML-GRM model implemented in GCTA. “REML-PED” is the relative residual variance and DRP variance calculated by fitting pedigree relationships with the REML-PED model implemented in DMU. “REML-PEDGRM” is the relative residual variance and DRP variance calculated by fitting both 50 k SNPs and pedigree relationships with the REML-PEDGRM model implemented in DMU. Results for the model that did not converge for the trait are not presented. [file 12711_2017_336_MOESM4_ESM.docx]

**Table S4 The Akaike information criterion (AIC) for different models compared to REML-PED**

| Traits and scenarios | GREML-MS | | | REML-GRM | REML-PEDGRM |
| --- | --- | --- | --- | --- | --- |
| YIELD | -1228.3 | | | -1163.6 | -1274.2 |
| MILK |  | -1678.8 |  | -1336.5 | -1459.4 |
| PROT |  | -1135.6 |  | -1188.1 | -1332.9 |
| FAT |  | -1424.3 |  | -1450.3 | -1565.9 |
| MILKORG |  | -251.4 |  | -249.8 | -263.9 |
| MILKSP |  | -917.8 |  | -865.0 | -950.2 |
| LONG |  | -135.3 |  | -87.6 | -261.9 |
| MASTI |  | -822.9 |  | -810.5 | -879.4 |
| HEALTH |  | -40.4 |  | -12.8 | -198.4 |
| LEG |  | -398.6 |  | -392.9 | -423.9 |
| CALV |  | -321.8 | | -307.8 | -346.5 |
| BIRTH |  | -531.0 |  | -505.4 | -538.5 |
| FERT |  | -349.2 |  | -345.2 | -469.8 |
| BODY |  | -573.1 |  | -563.8 | -570.4 |
| GROWTH |  | -1039.7 |  | -1046.0 | -1116.7 |
| TEMP |  | -76.1 |  | -73.2 | -145.5 |
