# Supplementary figures and images for: Contribution of rare and low-frequency whole-genome sequence variants to complex traits variation in dairy cattle
Source: Genet Sel Evol. 2017 Aug 1;49:60. doi: 10.1186/s12711-017-0336-z (PMC5539983; doi:10.1186/s12711-017-0336-z)

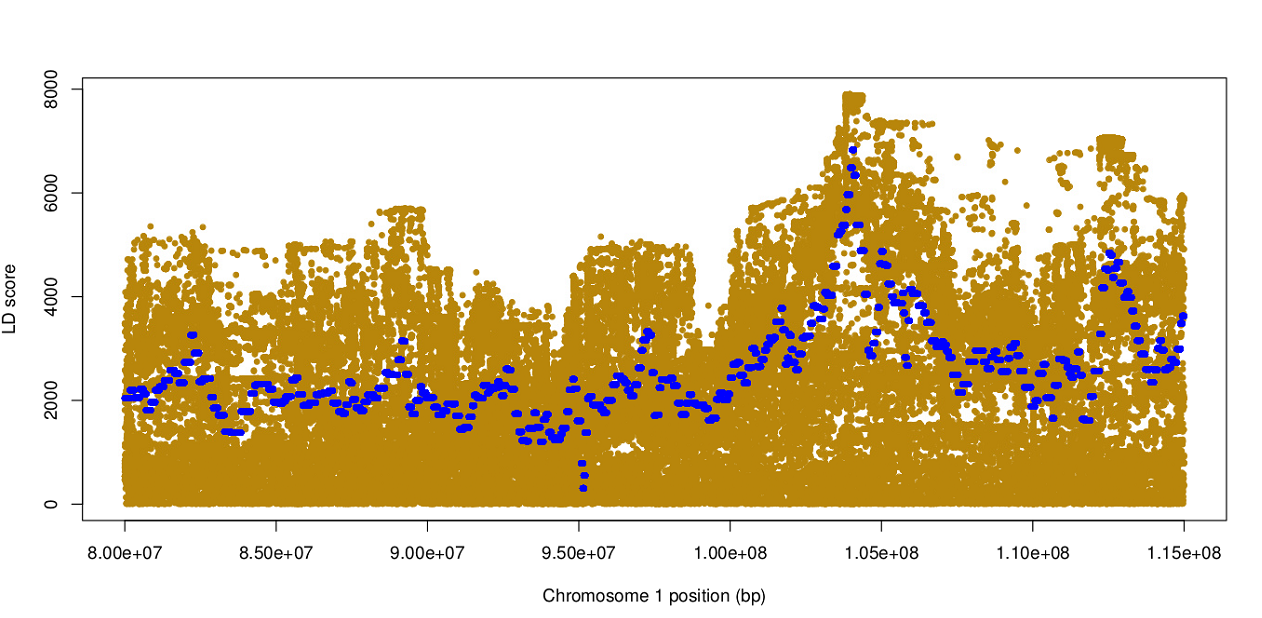

Supplement: Supplementary file 5 — Additional file 5: Figure S1. LD score on a segment of chromosome 1 in a sample of Holstein individuals. The yellow dots are the LD score for each variant. The LD score was defined as the sum of the LD measure r2 between this SNP and other SNPs in a 20-Mb region centered on this SNP. The blue dots are the average LD score for a sliding window of 100 kb. [file 12711_2017_336_MOESM5_ESM.tif]
